# Identification of *CETP* as a molecular target for estrogen positive breast cancer cell death by cholesterol depleting agents

**DOI:** 10.18632/genesandcancer.122

**Published:** 2016-09

**Authors:** Luke Esau, Sunil Sagar, Dhinoth Bangarusamy, Mandeep Kaur

**Affiliations:** ^1^ King Abdullah University of Science and Technology (KAUST), Computational Bioscience Research Center, Thuwal, Kingdom of Saudi Arabia; ^2^ King Abdullah University of Science and Technology (KAUST), Bioscience Core Lab, Thuwal, Kingdom of Saudi Arabia; ^3^ School of Molecular and Cell Biology, University of the Witwatersrand, Private Bag 3, Wits, Johannesburg, South Africa

**Keywords:** CETP, cholesterol depletion, breast cancer, acetyl plumbagin

## Abstract

Cholesterol and its metabolites act as steroid hormone precursors, which promote estrogen receptor positive (ER+) breast cancer (BC) progression. Development of cholesterol targeting anticancer drugs has been hindered due to the lack of knowledge of viable molecular targets. Till now, Cholesteryl ester transfer protein (CETP) has been envisaged as a feasible molecular target in atherosclerosis, but for the first time, we show that *CETP* contributes to BC cell survival when challenged with cholesterol depleting agents. We show that MCF-7 *CETP* knockout BC cells pose less resistance towards cytotoxic compounds (Tamoxifen and Acetyl Plumbagin (AP)), and were more susceptible to intrinsic apoptosis. Analysis of differentially expressed genes using Ingenuity Pathway Analysis (IPA), *in vivo* tumor inhibition, and *in vitro* phenotypic responses to AP revealed a unique *CETP*-centric cholesterol pathway involved in sensitizing ER+ BC cells to intrinsic mitochondrial apoptosis. Furthermore, analysis of cell line, tissue and patient data available in publicly available databases linked elevated *CETP* expression to cancer, cancer relapse and overall poor survival. Overall, our findings highlight *CETP* as a pharmacologically relevant and unexploited cellular target in BC. The work also highlights AP as a promising chemical entity for preclinical investigations as a cholesterol depleting anticancer therapeutic agent.

## INTRODUCTION

Clinical and experimental investigations evidence an unquestionable role for cholesterol in cancer progression [1]. This link is supported by multiple studies documenting levels of HDL and LDL in cancer patients [2–4], enhanced cholesterol synthesis in cancer cells [5–7], cholesterol accumulation in lipid rafts [8, 9], steroidogenesis in cancer cells [10, 11], over-expression of cholesterol transport receptors: SR-B1 (HDL) and LDL-receptor [12, 13], and more recently, the involvement of the cholesterol metabolite 27HC [14–16]. Thus the precedent has been set for developing cholesterol-modulating anticancer agents [17–19].

Breast cancer (BC) is a complex disease driven by lifestyle, genetics, and the abnormal function of hormones [20]. Dietary cholesterol [13] and particularly lipoproteins (both LDL and HDL) have been shown to fuel growth of breast tumours [20, 21], therefore a better understanding of cholesterol homeostasis for future BC therapeutics is necessary. The cholesteryl ester transfer protein (CETP) shuttles cholesteryl esters (CEs) and triglycerides between HDL and LDL to maintain cholesterol homeostasis both intracellularly and extracellularly [22]. CETP has been shown to prevent development of atherosclerotic lesions in ovariectomized hypercholesterolemic mice [23],and a link between CETP and estrogen has also been proposed [24]. Furthermore, CETP-deficient cells have been reported to have lower cholesterol content as CETP deficiency impeded CE transport from its site of synthesis to the storage droplet thereby reducing CE hydrolysis [25]. Inhibiting CETP was envisaged as a viable strategy to prevent coronary heart disease [26], and despite leading to several failed clinical trials its role in cancer has been overlooked. This biological paradigm calls for re- evaluation of the biological functions of CETP in diseases.

In the present study, we present a small molecule, acetyl plumbagin (AP), with cholesterol modulatory features and evidence of *CETP* as a cell survival gene that facilitates BC proliferation and resistance to apoptosis.

## RESULTS

### *In vitro* and *in vivo* anticancer effects of AP

**Figure 1:**
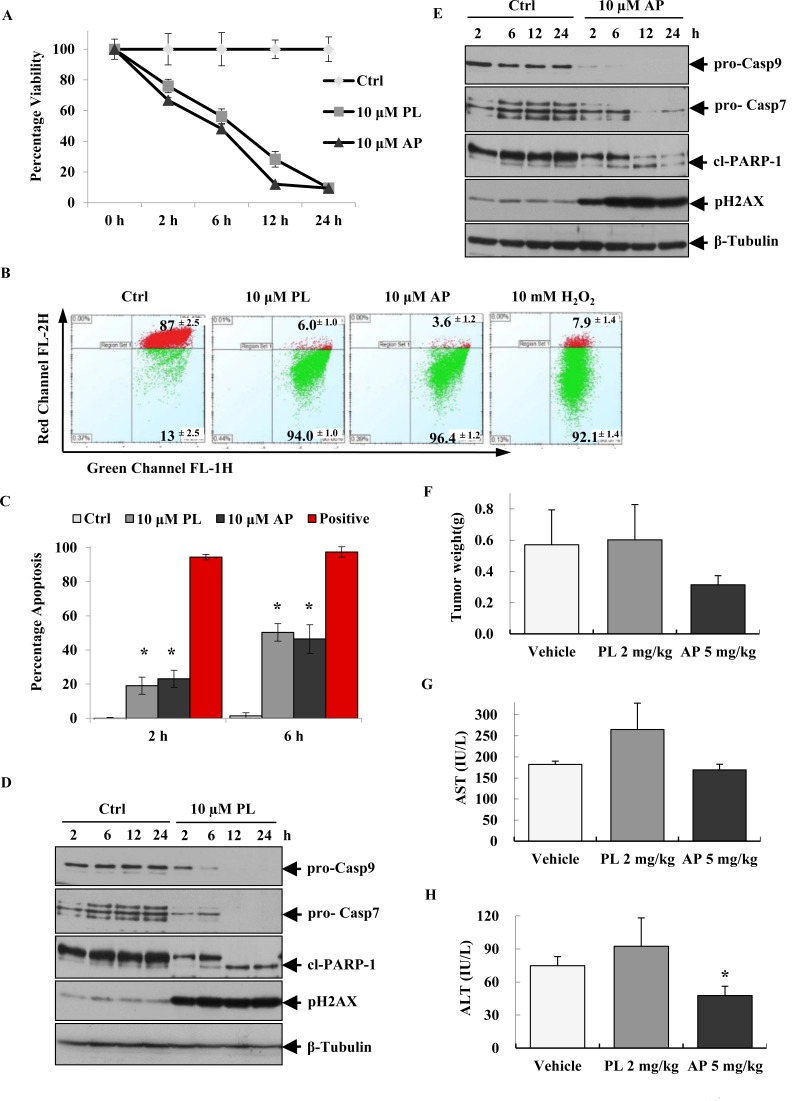
AP induces intrinsic apoptosis in MCF-7 cells and reduces tumour burden in MCF-7 xenograft models (A) Viability of MCF-7 cells over time after treatment with 10 μM PL and AP. (B) MOMP disruption as measured by flow cytometry after treating MCF-7 cells with 10 μM PL and AP for 1 h; Ctrl represents cells treated with 0.2% DMSO. (C) The apoptosis-inducing potential of 10 μM PL and AP in MCF-7 as determined by using APOPercentage assay performed at 2 and 6 h. Data shown (in A, B, and C) are representative of mean ± SD of quadruplicate wells/condition in at least two independent experiments (n=2).* Indicates P<0.05 as calculated by students t-test. (D-E) Western blot analysis of pro-Casp9, pro-Casp7, PARP-1 and pH2AX after incubating MCF-7 cells for 2, 6, 12, and 24 h with PL and AP. β-Tubulin was used as a loading control. Images are representative of three independent experiments (n = 3). (F) The *in vivo* effect of PL (2 mg/kg) and AP (5 mg/kg) on tumour weight and plasma levels of (G) AST and (H) ALT in different groups of MCF-7 xenograft models after 21 days drug treatment. Data shown (in F, G and H) as mean ± SD of vehicle group (n = 4), PL- treated (n = 4) and AP-treated (n = 5) mice.

Our previous study highlighted AP (a derivative of PL) as a potential anticancer lead molecule [27]. AP was selected for further *in vitro* and *in vivo* investigations based on key characteristics including: its low *in vitro* toxicity towards normal skin fibroblast BJ cells and its selective activation of caspase and apoptosis pathways in MCF-7 cells compared to BJ and triple negative BT20 cells. The chronology of apoptosis induction was determined by measuring cell viability, mitochondrial outer membrane potential (MOMP), apoptosis staining and apoptosis markers by western blot analysis in MCF- 7 cells treated with AP (PL was used as the parent drug control). MCF-7 viability was reduced as early as 2 h and continued to decrease with time in response to PL and AP treatment (Figure 1A). Consistent with reduced viability, we observed MOMP collapse within 1 hour (Figure 1B) and apoptosis induction at 2 and 6 h (Figure 1C). Western blot of pro-caspases 9 and 7, the DNA damage marker pH2Ax and cl-PARP also suggested the involvement of the intrinsic apoptotic pathway in MCF-7 cell death (Figure 1D and 1E). These findings provided a time frame for further *in vitro* experiments. In addition, MCF-7 xenograft mouse models treated with 5 mg/kg AP for 21 days, displayed a 45% reduction in tumour weight, while 2 mg/kg PL treatment exerted no observable effects (Figure 1F). Generally, elevated plasma concentrations of the liver enzyme markers alanine transaminase (ALT), and aspartate transaminase (AST), present in serum or blood, are indicative of stress and toxicity. While PL treatment increased the activity of AST and ALT enzymes in mice serum (Figure 1G and 1H), AP reduced ALT and AST activity by 36% and 7% respectively, as compared to vehicle. It is worthwhile mentioning that mice were initially treated with 5 mg/kg PL however within two days 8 mice succumbed to treatment hence dosage was adjusted to 2 mg/kg. Although *in vitro* data suggested similar mechanisms of action for PL and AP, which is not unusual as these compounds only differed by a methoxy group, *in vivo* results suggested otherwise i.e. AP and PL may possess alternate mechanisms since the toxicity of AP *in vivo* was substantially lower as compared to PL.

### Identification of pathways affected by AP and PL

Next we wanted to elucidate pathways altered by PL and AP in MCF-7 cells to further understand phenotypic differences observed *in vivo*. MCF-7 cells treated with 10 μM PL or AP for 6 h were subjected to microarray analysis and differentially expressed genes were mapped to biological pathways contained in the IPA (Figure S1). Both PL and AP affected multiple pathways however a clear distinction could be drawn for PL which targeted pathways including ELF2 signalling (P = 2.04E-08), mTOR signalling (P = 7.57E-03), and the regulation of elf4 and p70S6K (P = 2.24E-02) while AP affected pathways such as hepatic fibrosis (P = 7.88E- 05), PXR/RXR activation (P= 3.86E-03), atherosclerosis signalling (P = 2.33E-02), and androgen biosynthesis (P = 1.52E-02) (Figure S1). Amidst the pathways altered, two distinguishing pathways emerged to which most PL and AP genes were mapped (Figure S2 and S3). For PL, a demarcated mapping of downregulated Complex I, III and IV genes involved in mitochondrial oxidative phosphorylation was noted. Evidence in literature for PL and mitochondrial related processes validate this observation [28–31]. AP altered apolipoprotein genes which mapped well to the atherosclerosis pathway.

### Validation of cholesterol depletion mediated cell death induction by AP in MCF-7 cells

**Figure 2:**
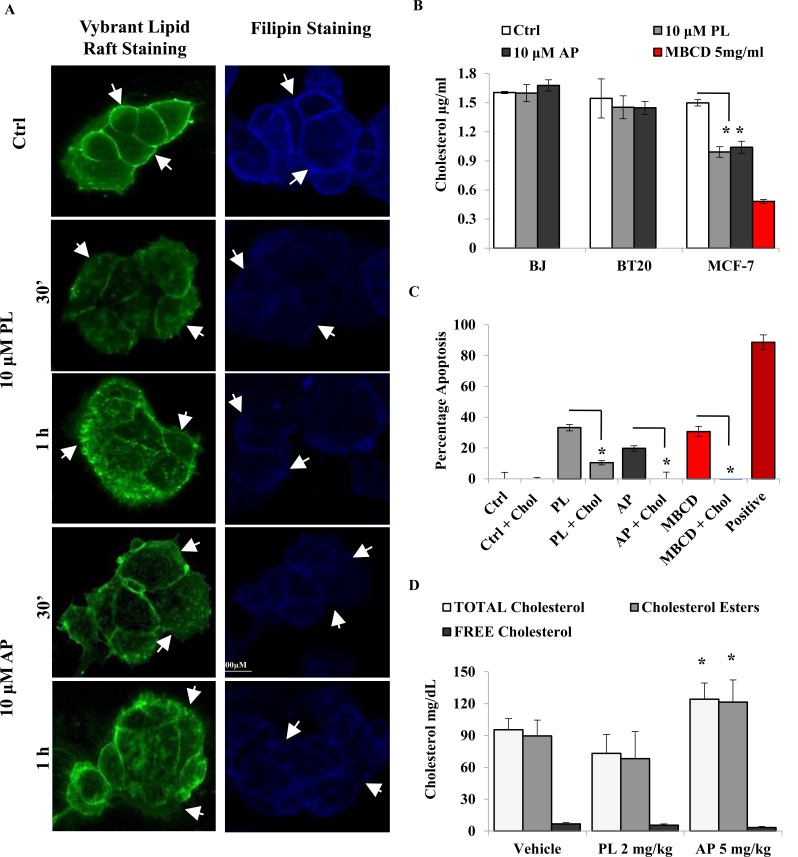
AP disrupts lipid rafts and modulates cholesterol levels *in vitro* and *in vivo* (A) Lipid raft and filipin staining in MCF-7 cells after treatment with 10 μM PL or AP for 30 min and 1 h. Images are representative of three independent experiments (n = 3). (B) Comparison of cholesterol depletion in BJ, BT20, and MCF-7 cells after treatment with 10 μM PL and AP for 2 h; 5 mg/ml MBCD was used as a positive control. (C) Cholesterol pre-incubation blocked apoptosis induction in 10 μM PL and AP, and 5 mg/ml MBCD treated cells for 2 h. 5 mMH_2_O_2_ was used as a positive apoptotic control. Data shown (B and C) are representative of the mean ± SD of quadruplicate wells/ condition in at least three independent experiments (n=3) (P ≤ 0.05, *t*-test). (D) Levels of total cholesterol, cholesterol esters, and unbound free cholesterol. Data shown as mean ± SD of vehicle group (n = 4), PL treated (n = 4) and AP treated (n = 5) groups.

Apolipoproteins are important components of the cholesterol pathway especially in the formation of HDL and LDL cholesterol. Furthermore cholesterol metabolism and BC aggressiveness and progression are intimately linked as evidenced in numerous studies [6, 7]. To validate cholesterol alterations suggested by our pathway analysis, we stained lipid rafts and cholesterol with the Vybrant Lipid Raft kit and Filipin respectively in cells treated with 10 μM PL or AP (Figure 2A and Figure S4A). The distinct membrane staining of lipid rafts and cholesterol (white arrows) observed in control cells was disrupted in MCF- 7 cells while BJ cells remained unaffected. Quantitative measurement of cholesterol in BJ, BT20 and MCF-7 cells by AMPLEX Red assay after 2 h PL and AP treatment revealed a 35% reduction of cholesterol in MCF-7 cells (Figure 2B). Neither BJ nor BT20 cells were depleted of cholesterol which could explain our previous observation regarding AP selectivity for ER positive BC cells [27]. Additionally cholesterol pre-treatment significantly reduced apoptosis in MCF-7 cells treated with PL (from 30% to 10%) and AP (from 20% to negligible) (Figure 2C), which is similar to observations reported [9]. This data suggests that AP induced cholesterol depletion which lead to increased apoptosis in MCF-7 cells. A significantly increased level of CEs and decreased free cholesterol was observed in mouse serum in the AP treated group of mice as compared to the vehicle and PL group (Figure 2D). The increase in CEs was in agreement with elevated HDL and LDL levels observed in AP treated mice (Figure S4B). Of more relevance is a physiological balance of total cholesterol/HDL and HDL/LDL ratios as an imbalance correlates with atherosclerosis progression and BC risk [32]. Total cholesterol/HDL and HDL/LDL ratios measured in vehicle and AP groups were concordant with published data while PL treatment disturbed the HDL/ LDL ratio in the PL group (Table 1).

**Table 1:** The Ratio of Total Cholesterol/HDL and HDL/LDL in mice

Study Group	Total Cholesterol/HDL	HDL/LDL
Reference	1.38	3.0
Vehicle	1.40	3.4
PL 2 mg/kg	1.3	1.8
AP 5 mg/kg	1.4	3.1

Ratios of total cholesterol: HDL and HDL: LDL/VLDL in mice serum. Values represent the ratios of means of the vehicle group (n = 4), PL-treated (n = 4) and AP-treated (n = 5) groups. Reference values are adapted from http://www.abcam.com/hdl-and-ldlvldl-cholesterol-assay-kit-ab65390.html.

### Identifying role of CETP in cancer survival and response to anticancer agents

**Figure 3:**
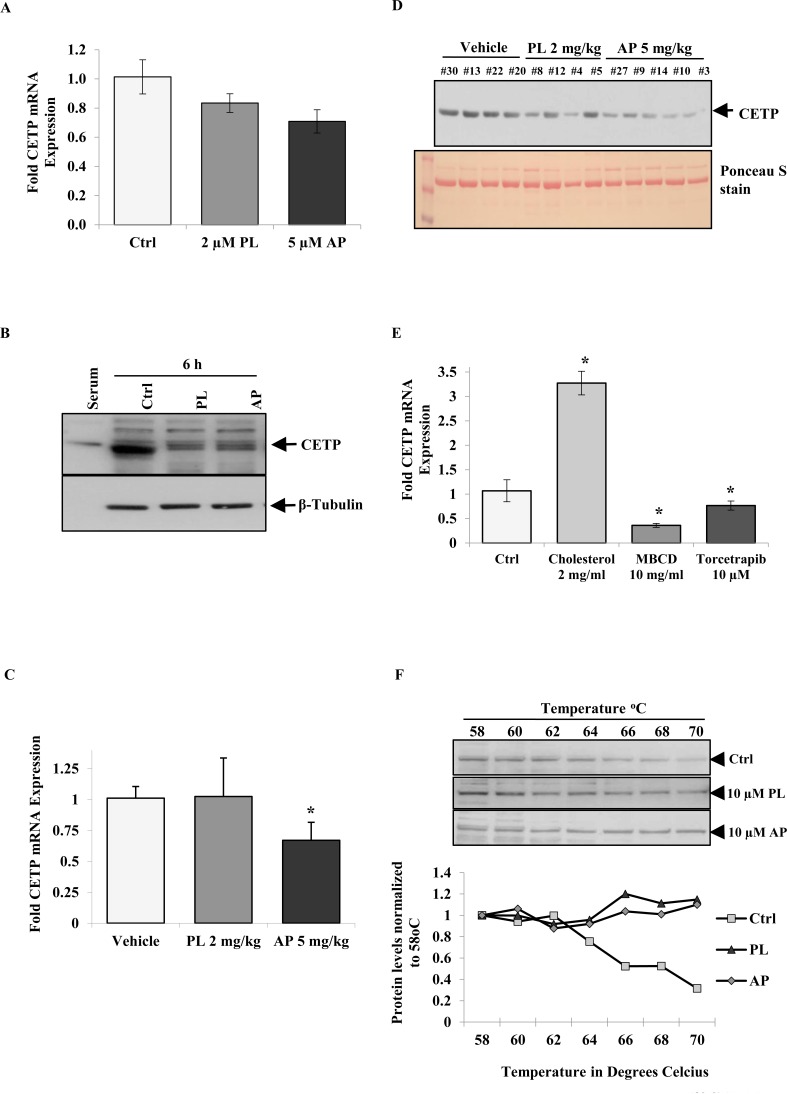
*CETP* expression is altered in response to cholesterol modulation Levels of (A) *CETP* mRNA in MCF-7 cells treated for 5 days with 2 μM PL and 5 μM AP and CETP protein levels when treated with 10 μM PL and AP for 6 h (B). *CETP* mRNA expression in tumour samples (C) and CETP protein levels in mice serum samples (D) after 21 days treatment with PL (2 mg/kg) and AP (5 mg/kg). (E) MCF-7 cells treated with 2 mg/ml Cholesterol, 10 mg/ml MBCD and 10 μM Torcetrapib for 48 h. Data shown (C and E) are mean ± SD of quadruplicate wells of three independent pooled experiments (P ≤ 0.05, *t*-test). Comparison of CETP protein stability in Ctrl and PL or AP-treated MCF-7 cells during cellular thermal shift analysis. Data shown are representative of three independent experiments (F).

Key proteins involved with regulating cholesterol homeostasis are pertinent to processes including reverse cholesterol transfer (RCT), steroid hormone production and atherosclerosis among others [26, 33]. CETP shuttles CEs and triglycerides between plasma lipoproteins and plays a vital role in reciprocal transfer of these from LDL/ VLDL to HDL. Advanced data mining also connected CETP to the atherosclerosis pathway, which led us to investigate the role of *CETP* in MCF-7 cells (Figure S5). Treatment with low concentrations of PL and AP for five days’ decreased the *in vitro* expression of *CETP* mRNA by 15% and 33% respectively (Figure 3A). CETP protein level was also reduced after 6 h treatment with PL and AP (Figure 3B). A significant (35%) reduction of *CETP* mRNA was observed in tumour RNA isolated from mice treated for 21 days with AP however PL treatment had no effect (Figure 3C). Furthermore western blot detection revealed reduced CETP serum levels in two out of four PL treated mice and five out of five AP treated mice (Figure 3D). In light of our observations of altered cholesterol metabolism (Figure 2), CETP mRNA and protein levels seem to fluctuate in response to cholesterol levels, which has been reported in literature. In a study by Mark et al [34], CETP levels were upregulated in liposarcoma cells SW872 when incubated with cholesterol. Tissues including hepatocytes and luminal epithelial cells are also known sources of CETP expression however *CETP* expression in response to stimulus has not been reported for BC. MCF-7 cells were incubated with cholesterol, a cholesterol depletory MBCD or the CETP inhibitor Torcetrapib for 48 h and *CETP* mRNA levels were determined (Figure 3E). Interestingly, *CETP* expression was significantly upregulated by cholesterol repletion (≥3 fold) while significantly downregulated (≤ 2 fold) by cholesterol depletion or *CETP* inhibition. Considering that Torcetrapib binds to CETP with high affinity and our observation that it down-regulated CETP expression, we explored by cellular thermal shift assay (CETSA) whether PL and AP potentially bound to CETP accounting for the decrease in *in vitro* and *in vivo* CETP mRNA and protein levels (Figure 3F). Although CETSA is a crude method for measuring protein-drug interactions, we show that PL and AP were able to substantially increase the thermal stability of CETP compared to control suggesting a potential for CETP-PL/AP interactions.

**Figure 4:**
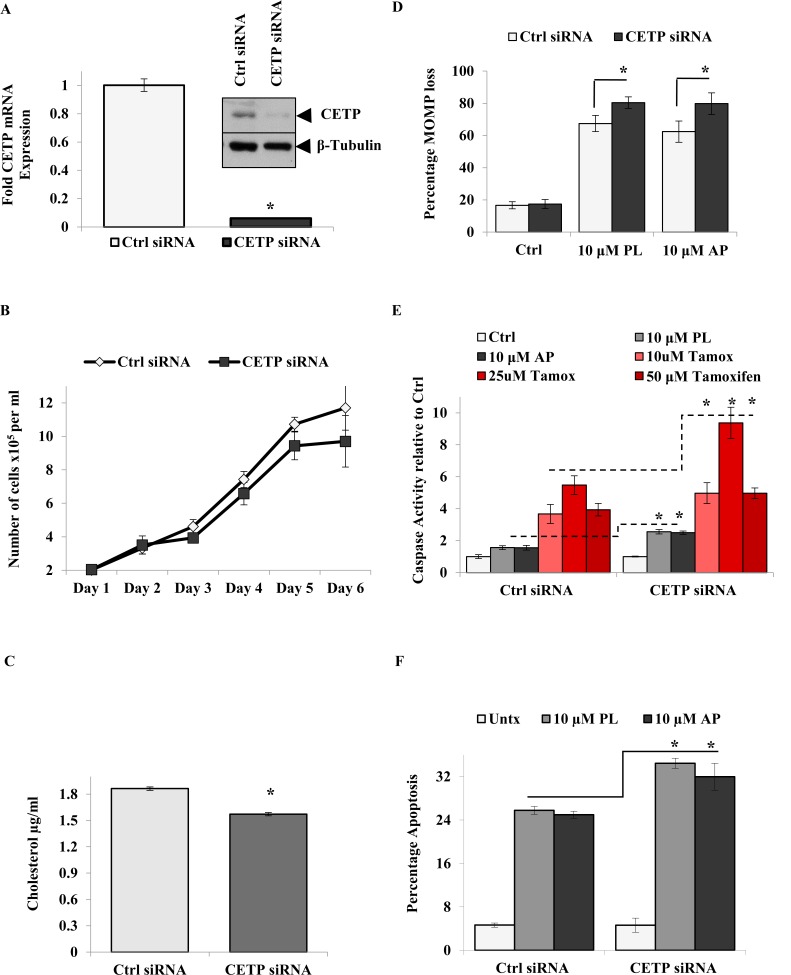
*CETP* contributes to cancer cell growth and survival and is upregulated in breast cancer patients (A) *CETP* gene silencing by siRNA and western blot analysis of CETP protein expression in *CETP* siRNA cells. (B) Effect of *CETP* mRNA silencing on growth of MCF-7 cells as measured by cell counting over a period of six days. (C) Comparison of cholesterol levels in the MCF-7 control (ctrl) siRNA and *CETP* knockout cells. Data shown are the mean ± SD of quadruplicate wells/condition in three independent experiments (n = 3) (P ≤ 0.05, *t*-test). (D) The effect of 10 μM PL or AP treatment on MOMP; (E) Capase-3/7 activity (10, 25 and 50 μM Tamoxifen was used as a positive control); and (F) apoptosis in MCF-7 ctrl siRNA and *CETP* siRNA knockout cells. Data shown are representative of the mean ± SD of quadruplicate wells/condition in at least two independent experiments (n=2) (*P ≤ 0.05, *t*-test).

We next determined the effect of *CETP* ablation on MCF-7 cell proliferation and survival. Torcetrapib significantly reduced cell growth while not inducing apoptosis at all concentrations tested (Figure S6A and B). Employing a more specific approach using a siRNA cocktail directed to *CETP* mRNA, CETP gene and protein expression was significantly decreased in MCF-7 cells (Figure 4A). *CETP* knockdown also decreased cellular growth after 6 days in MCF-7cells (Figure 4B) while only transiently on days 4 and 5 in BJ cells (Figure S6C).

Cholesterol is important for the synthesis of new membranes in dividing cells and is assimilated into lipid rafts, which provide a scaffold for proliferative receptors (e.g. EGFR) and survival factors (e.g. Akt) [9, 35]. Interestingly, CETP was shown to increase cholesterol uptake in HepG2 cells [36]. To this end, we measured the effect of *CETP* mitigation on cell cholesterol content and apoptosis in BC and normal BJ cells. *CETP* gene silencing resulted in a significant reduction in cellular cholesterol by 20% which further antagonized MOMP loss in the presence of PL or AP (Figure 4C and 4D). In conjunction with increased disruption of MOMP, caspase activity and apoptosis were significantly upregulated in *CETP* siRNA cells compared to Ctrl siRNA cells in response to PL, AP or Tamoxifen treatment (Figure 4E and F). However, BJ cells treated with *CETP* siRNA were not further sensitized to PL or AP treatment (Figure S6D). Although not significant, Casp 7 mRNA was also upregulated in *CETP* knockout cells which possibly contributed to the observed increase in drug sensitivity (Figure S6E).

**Figure 5:**
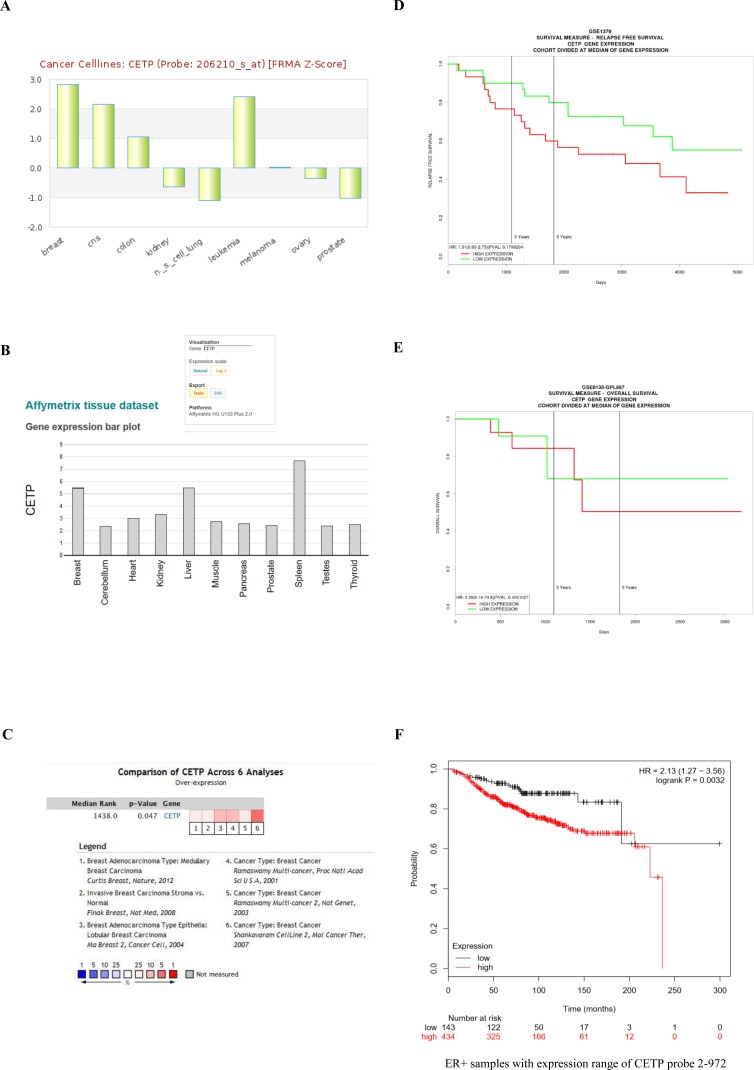
*CETP* expression analysis in BC cell lines and patients The expression of *CETP* in three different databases: (A) the expression of *CETP* in NCI panel of 60 cell lines (http://medicalgenomics.org/details_view_limited?db=cellnavigator&gene_id=1071&cn_option=m_disease#v), (B) the expression of *CETP* in BC tissue in the Affymetrix tissue dataset (http://www.betastasis.com/tissues/affymetrix_tissue_dataset/gene_expression_barplot_affymetrix_hg_u133_plus_20/), and (C) shows expression of *CETP* in six different BC patient datasets in the Oncomine database. (D and E) Kaplan-Meier plots of relapse-free survival and overall survival of BC patients stratified by median *CETP* expression in the Proggene database [48]. (F) the effect of *CETP* expression level on survival of ER+ and ER- BC patients estimated using KM-Plotter database. Log-rank test *P*-value is displayed on the graphs.

Thus far our data highlighted key roles of *CETP* in BC survival and growth linked to cholesterol metabolic pathways. To assess the relevance of variations in *CETP* expression, we analysed several online available microarray based BC databases. We found using the Cell Navigator tool, among several cell lines frequently used by NCI for anticancer drug testing, elevated CETP expression in BC cell lines (Figure 5A). The expression of *CETP* was also higher in BC tissue as compared to other tissues available in the Affymetrix tissue dataset (Figure 5B). Furthermore the expression analysis showed significantly elevated *CETP* expression in BC vs normal patients in six previously published datasets contained in Oncomine (Figure 5C). Correlation of *CETP* expression with BC patient survival using four different BC datasets revealed that patients expressing low levels of *CETP* have longer relapse free survival and improved overall survival (Figure 5D and E). Lastly we found, using the online KM- Plotter database [37], that ER+ BC patients express higher levels of *CETP* and have a significantly lower probability of long-term survival (P-value < 0.0032) as compared to ER- patients (Figure 5F). While compiling our results an indirect evidence for involvement of *CETP* in the aetiology of BC in African American women came from a study base on gene-based and single-SNP analyses [38].

## DISCUSSION

The advent of linking cholesterol, cholesterol metabolites and cholesterol modulators with BC is changing the view for future BC management and treatment. Altogether a better understanding of cholesterol processes in ER+ BC is being unravelled in light of failed clinical response to selective ER modulators (SERMs) like Tamoxifen and aromatase inhibitors. Recently Shim et al. found that endothelial and BC cell proliferation was inhibited in a cholesterol-dependent manner, unrelated to ER inhibition, by high concentrations of Tamoxifen [39]. Notably continued activation of ER signalling despite aromatase therapy or tamoxifen treatment has been attributed to non-aromatized molecules like 27 hydroxycholesterol (27HC) and 25 hydroxycholesterol (25HC). Wu et al. reported that ER+ BC cell growth was augmented both *in vitro* and *in vivo* by 27HC [16]. Furthermore the abundance of 27HC was found elevated in normal breast tissue and in the adjacent cancer site of BC patients [16]. Likewise, 25HC stimulated MCF- 7 and BG-1 cell growth via the ERα *in vitro* and 25HC plasma levels were increased after a high cholesterol meal *in vivo* [40]. Interestingly the expression of oxysterol 7α-hydroxylase (CYP7B1), an enzyme which catabolises both 27HC and 25HC, is decreased in ER+ BC patient tissue and is predictive of overall poor survival [16, 40]. The cholesterol pathway constituents like LDL cholesterol, cholesterol receptors LDLR and SCARB1 and others have strong correlation with BC progression and clinical outcome [15, 21, 41–44]. These emerging data undeniably highlights the requirement for cholesterol modulating agents in BC management.

Although PL has been well studied for over 40 years, its failure in clinical testing due to toxicity may have impeded the discovery of PL derivatives, such as AP, as anticancer lead molecules. In our current study we provide intriguing evidence identifying *CETP* as a potential molecular target in ER+ BC. We found that AP induced apoptosis *in vitro* and was able to reduce tumour growth *in vivo* with no associated toxicity. Gene expression analysis was further able to delineate ROS and atherosclerosis pathways targeted by PL and AP respectively previewing their mechanisms of action. We further confirmed, as predicted by pathway analysis, that AP is a cholesterol depletor as evidenced by *in vitro* and *in vivo* findings. Despite PL displaying similar *in vitro* characteristics to AP, its dissonance with *in vivo* mechanisms may be explained by its characteristic ROS inducing properties. ROS accumulation potently oxidizes proteins, lipids and causes DNA damage which we observed previously and in this study [27]. Furthermore HDL, being one of the key transporters of hydroperoxides to hepatocytes, is more prone to lipid peroxidation during major disruptions in the redox status. An *in vivo* study by Sukkasem et al. is one of few studies reporting an imbalance in the anti- oxidative system and hepatotoxic effects of PL in mice [37]. Similar to this study, we also observed an increase in the liver damage markers ALT and AST in mice plasma and substantial alterations of the HDL/LDL ratio in the PL treated mice group all of which may have been due to PL induced ROS build-up. AP on the other hand significantly increased cholesterol and HDL/LDL levels in serum without disturbing the HDL/LDL ratio suggesting increased cholesterol efflux. Although AP is capable of producing ROS, its unique mechanism of action as determined by pathway analysis and *in vivo* phenotype suggests a distinct interactome. In retrospect these factors may underpin the *in vivo* differences seen for AP and PL.

The highlight of our findings is that AP, decreased *CETP* gene expression and protein levels *in vitro* and in MCF-7 BC xenografts. For the first time, we also show alteration of *CETP* gene expression in MCF-7 cells in response to: Torcetrapib treatment; to addition or depletion of cholesterol and binding of CETP to AP by CETSA which may provide insights into the regulation of CETP. Importantly we show that *CETP* contributes to MCF-7 proliferation, cholesterol uptake and survival as silencing *CETP* reduced cell numbers, cholesterol content and sensitized cells to apoptosis. Our analysis of *CETP* expression in several databases across cell line, BC tissue and patient gene expression data also provides evidence for the implication of *CETP* in BC as it was found commonly overexpressed in BC cases, correlated with poor relapse free and overall survival and a significant probability of poor long-term survival for ER+ BC patients. These observations may be crucial in light of the evidence that *CETP*: is elevated in BC; increases cholesterol uptake in BC cells; mediates the transfer of free cholesterol between lipoproteins and membranes; shuttles estradiol (E2) between HDL and LDL; and augments estradiol delivery to tissues, potentially enhancing estrogen signalling [24]. Furthermore, AP significantly decreased estrogen receptor– 1 (ESR-1) and elevated the *CETP* inhibitor APOF gene expression which further adds another level of regulation mediated by the therapeutic small molecule (AP) in the *CETP* pathway (Figure S6F and G). AP may have potential as an anticancer drug and anti-atherosclerosis agent. The present study lacks testing of *CETP* knock-out on cholesterol processing and storing capacity of the BC cells, and also molecular effects of *CETP* knock-out on HDL/LDL sizes and expression of various receptors involved in the cholesterol pathway. Nevertheless, our findings provide evidence for a previously unknown role of *CETP* in ER+ BC which may present a pharmacologically important and unexploited cellular target in BC. Further in-depth investigation of *CETP*'s contribution in developing and sustaining BC growth is warranted.

## MATERIALS AND METHODS

### Materials

Dulbecco's Modified Eagle Medium (DMEM), fetal calf serum (FCS), penicillin-streptomycin, Trizol, propidium iodide (PI), and TaqMan Universal Master Mix were obtained from Life Technologies, UK. Tamoxifen, paclitaxel, methyl-beta-cyclodextrin (MBCD), hydrogen peroxide (H_2_O_2_), cholesterol, 5-fluorouracil, PL, and MTT (3-(4,5-Dimethylthiazol-z-yl)-2,5-diphenyltetrazolium bromide) were purchased from Sigma Chemicals, USA. AP was synthesized as previously described [27].

### Cell Culture

Authenticated by STR profiling and mycoplasma-free MCF-7 (human breast adenocarcinoma; ATCC^®^ HTB-22TM), BT20 (human breast carcinoma; ATCC^®^ HTB-19 TM), and BJ cells (normal skin fibroblasts; ATCC® CRL-2522 TM) were procured from the American Type Cell Culture Collection (ATCC, Manassas, VA). Cells were cultured in DMEM supplemented with 10% FCS, penicillin (100U/ml), and streptomycin (100 μg/ml) (Gibco) at 5% CO2 in a 37°C incubator.

### Growth inhibition assay

The effect of PL and AP on cell viability was estimated by using the MTT assay as previously described [27], control cells were treated with relevant concentrations of solvent only (maximum 0.2% DMSO). Optical Density was measured at 595 nm using a microtiter plate reader (BMG Labtech PHERAstar *FS*, Germany), and the percentage viability was determined using Microsoft Office Excel©.

### APOPercentage Assay

MCF-7 cells seeded in 96-well plates were treated with 0.2% DMSO (control), PL and AP compounds for the times described previously [27]. Cells were detached and stained with APOPercentage dye (Biocolor, UK) as described in [45], and apoptotic cells were quantified with a high throughput flow cytometer (HTFC) screening system (IntelliCyt Corporation, Albuquerque, NM).

### Mitochondrial Outer Membrane Potential (MOMP) assay

MCF-7 cells cultured in 96-well plates were stained with 2μM cyanine dye JC-1 (5,5′,6,6′-tetrachloro-1,1′,3,3′- tetraethylbenzimi- dazolylcarbocyanine iodide) (Life Technologies, UK) for 1 has described [45]. 10mM H_2_O_2_ was used as a positive control and cells were analysed by the HTFC system by plotting FL2-H vs. FL-1H and applying a quadrant gate to determine JC-1 aggregates (red) and monomers (green).

### RNA isolation and cDNA synthesis

As previously described [27], total RNA isolated from MCF-7 cells was converted to cDNA and stored at −20°C. The tumour tissue samples frozen in liquid nitrogen were placed into cold tubes in a MasterPrepTM24 (Hichuang Co.) and homogenized. Trizol (1 ml/50-100 pellet and the tube was centrifuged at 7500 × g for 5 min at 4°C. After pouring off the supernatant, the RNA pellet was resuspended in 30 μl of RNase-free water, and was stored at −80°C. mg tissue) was added to the tissues, and homogenized samples were kept on ice. After homogenization, the samples were incubated at room temperature for 5 min, 200 μl of chloroform was added to each sample, and sample was vigorously shook by hand for 15 seconds and incubated at room temperature for 2–3 minutes (min). Samples were centrifuged at 12,000 × g for 15 min at 4°C, and the aqueous phase was transferred to a new 1.5 ml microcentrifuge tube. Next, 0.5 ml of isopropanol was added to the aqueous phase, incubated at room temperature for 10 min, and centrifuged at 12,000 × g for 10 min at 4°C. The supernatant was removed, leaving only the RNA pellet. 1 ml 75% ethanol was added to the pellet and the tube was centrifuged at 7500 × g for 5 min at 4°C. After pouring off the supernatant, the RNA pellet was resuspended in 30 μl of RNase-free water, and was stored at −80°C.

### Gene Expression using qRT-PCR

Quantitative gene expression for *CETP* (Applied Biosystems TaqMan Assay No. s2934) was performed on a StepOnePlus™ Real-Time PCR machine (Applied Biosystems) using the TaqMan Universal Master Mix (Applied Biosystems). GAPDH was used as a housekeeping gene and fold gene expression was calculated using the 2^−ΔΔCT^ method.

### siRNA transfection

6 × 10^5^ cells were transfected with 40nmol*CETP* siRNA (Applied Biosystems) or Control siRNA (Eurofins) using the FuGene HD (Promega) reagent as per the manufacturer's instructions. Briefly, cells were trypsinized with 2 ml of trypsin, and 4 ml of complete media was added to deactivate the trypsin. Cell numbers were determined and 6 × 10^5^ cells were aliquoted into 2 ml of media. The transfection cocktail containing 30 μl of the FuGene HD and either 4 μl of the control or *CETP* siRNA (40 nmol) in 500 μl of serum-free media was added dropwise to cells. Cells were cultured in 6- or 96- well plates for 72 h before further analysis was performed.

### Caspase-3/7 activity

Cells were seeded at a density of 2.5 × 103 cells per well in 20 μl of media in 384-well plates and treated with PL and AP for the desired time. Caspase-3/7 activity was determined with the ApoTox-Glo kit (Promega) according to the manufacturer's instructions, and luminescence was measured using a plate reader (BMG Labtech PHERAstar *FS*, Germany).

### Lipid-Raft Staining

Cells grown in 6-well plates to 50-60% confluency were stained with the Vybrant® Alexa Fluor® 594 Lipid Raft Labeling Kit (Life Technologies) and Alexa Fluor® 594 Phalloidin (Life Technologies) according to the manufacturer's instructions. Images were captured with a Floid® Cell Imaging Station (Life Technologies).

### Cholesterol Assays

Cells plated at a density of 5 × 103 per well in a 96- well plate for 24 h were treated with AP/PL as indicated in the Figures. For cholesterol rescue experiments, cells were pretreated with 1 mM cholesterol (chloroform soluble) for 1 h followed by AP/PL treatment for apoptosis or with 2 mg/ml water soluble cholesterol for 48 h for *CETP* gene expression. Total cholesterol, HDL, and VLDL/ LDL levels were determined using the HDL and LDL/ VLDL Quantification Colorimetric/Fluorometric Kit from BioVision following the manufacturer's recommendations. Free cholesterol and cholesterol esters were measured using the Amplex® Red Cholesterol Assay Kit (Life Technologies) as per the manufacturer's instructions.

### Microarray and pathway identification

RNA was extracted as per the method previously described [27] and was quantified and quality checked using a nanodrop-ND 8000 spectrophotometer (Thermo Fisher Scientific, San Jose, CA, USA) and the 2100 Bioanalyzer (Agilent Technologies, Santa Clara, CA, USA), respectively. Samples with an RNA integrity number (RIN) of nine and above were chosen for microarray analysis, which was performed using the Human Gene Expression 4×44K v2 Microarray Kit, Design ID: 026652 (Agilent Technologies) containing 45220 features and 1377 Agilent control probes. The signal intensities were extracted from the scanned images with the Feature Extraction Software 10.7.1.1 (Agilent Technologies) and were subjected to background subtraction and spatial detrending. The outliers and the abnormal features were flagged, and the data was normalized using the intra-array percentile shift normalization (threshold of 75 and above) and median- based inter-array normalization. The GeneSpring GX (Agilent Technologies) was used to calculate the intensity ratios and fold changes. All the genes with a P < 0.05 and a fold change of 2 were chosen for pathway analysis. The pathways were generated through the use of QIAGEN's Ingenuity Pathway Analysis (IPA®, QIAGEN Redwood City, www.qiagen.com/ingenuity). Microarray data has been made publicly available in GEO (no: GSE68026).

### Xenograft study

The nonblinded study was performed by GenScript (http://www.genscript.com) with approvals of study protocols and animal use under IACUC No: 07X02015312.01. Initially ten mice per study group were randomly selected but eight mice succumbed to treatment due to toxicity within four to six days when treated with 5 mg/kg PL; hence the well-tolerated PL dose of 2 mg/kg was used (data not shown). We included four more mice in the PL treatment study. Body weight was used as an exclusion criterion to balance the number of mice in each group. Later, one mouse in the vehicle and two mice in PL-treated group also died.

BALB/c female nude mice were used for the study. MCF-7 cells (1.0×107) in 0.1 ml of PBS plus matrigel (1:1) /mouse were implanted, when tumours reached 300–500 mm3, the tumour mass was harvested, cut into 1–2 mm3 pieces, and then surgically implanted into the study group. Before the study began, tumours were allowed to reach 150–200 mm3. The mice were divided randomly into three groups: vehicle (n=4, 25% PEG400, 200μl/20g mouse, i.p., q.d.), AP treated (n=5, 5 mg/kg, i.p, qd), and PL treated (n=4, 2 mg/kg, i.p., q.d.), and treated for 21 days. At the end of the experiment the mice were necropsied; blood was collected for aspartate aminotransferase (AST) and alanine aminotransferase (ALT) measurements. Tumour volume and body weight were measured every second day. Tumour size was expressed in mm3 using the formula V = 1/2×a×b2, where ‘a’ and ‘b’ represent the long and short diameters of the tumour, respectively. The tumour mass was weighed, photographed after harvest and snap frozen in liquid nitrogen for RNA extraction. Tumour inhibition rate was calculated as inhibition rate (%) = (average tumour volume of control group-average tumour volume of test group)/ average tumour volume of control group×100%.

### Cellular Thermal Shift Assay (CETSA)

The protein-drug binding thermal shift assay was employed to test the binding of PL/AP to CETP [46]. Briefly MCF-7 cells grown to 60% confluency in T75 flasks were treated with PL, AP or Torcetrapib for 2 h at 37°C. Cells were trypsinized, counted, and washed twice with 1x PBS. The cell pellet was resuspended in 1 × EDTA-free protease inhibitors and aliquoted into PCR tubes at 1.5 × 10^6^ cells per tube. Tubes were placed in a Veriti® 96-Well Thermal Cycler (Applied Biosystems) and incubated for 3 min at 56–74°C with 2°C increments. Tubes were taken out of the thermal cycler and incubated at room temperature for 3 min. Cells were lysed by freeze-thawing the tubes twice in liquid nitrogen and at 25°C for 3 min with vortexing in between freeze-thaw cycles. Immediately after, cells were centrifuged at 14 000 rpm and 4°C for 20 min, and the supernatant was transferred to a clean Eppendorf tube. Protein was quantified using the BCA protein determination kit (Pierce Thermo Scientific, USA), and equal amounts of protein (15-20 μg) were subjected to Western blotting for CETP detection.

### Western Blotting

Protein lysate harvested from MCF-7 cells was quantitated with the BCA protein determination kit (Pierce Thermo Scientific, USA). An equal amount (20-30 μg) of protein lysate was subjected to gel electrophoresis on 10% SDS page gels, transferred to nitrocellulose membrane and probed with antibodies to PARP-1 (C2- 10, Trevigen, diluted 1:1000), Pro-Caspase-7 (PRS3467, Sigma, diluted 1:1000), Pro-Caspase-9 (MA1-12562, Thermo Scientific, diluted 1:1000), pH2AX (ADI-KAM- CC255, Enzo Life Sciences, diluted 1:4000), and CETP (PA1-050, ABR, diluted 1:500). β-Tubulin (sc-9104, Santa Cruz Biotechnology, diluted 1:1000) was used as a loading control. Fold change in expression of the serum protein was determined using densitometry analysis of Western blots using ImageJ.

### Statistical analysis

A Z-factor of ≥ 0.6 was recorded for each assay, indicating good to excellent robustness [47]. A student's *t*-test was used to determine statistical significance where p<0.05 or p<0.005. All statistics including mean and standard deviation (SD) calculations were performed using Microsoft Office Excel©. A normal distribution of data was assumed for statistical analyses. SD was calculated to estimate variation within each group of data and the range of SD was typically similar between groups. Experiments were performed in 96- and 384-well plates in duplicate/ triplicate as indicated in the Figure legends and each experiment further included triplicate or quadruplicate wells for each condition.

## SUPPLEMENTARY FIGURES



## Abbreviations

25HC: 25 Hydroxy-cholesterol
27HC: 27 Hydroxy-cholesterol
ALT: alanine aminotransferase
AP: Acetyl Plumbagin
AST: aspartate aminotransferase
BC: Breast cancer
CE: Cholesteryl ester
CETP: Cholesteryl ester transfer protein (protein)
*CETP*: Cholesteryl ester transfer protein (mRNA)
CYP7B1: oxysterol 7α-hydroxylase
DMEM: Dulbecco's Modified Eagle Medium
ER: Estrogen receptor
FCS: Fetal calf serum
HDL: High density lipoprotein
HTFC: High throughput flow cytometer
H_2_O_2_: Hydrogen peroxide
i.p.: *Intraperitoneal*
IPA: Ingenuity Pathway Analysis
JC-1: (5,5′,6,6′-tetrachloro-1,1′,3,3′-tetra ethyl benzimi- dazolylcarbocyanine iodide)
LDL: Low density lipoprotein
MBCD: Methyl-beta-cyclodextrin
MTT: (3-(4,5-Dimethylthiazol-z-yl)-2,5-diphenyl tetrazolium bromide)
NCI: National cancer institute
PBS: phosphate buffered saline
PI: Propidium Iodide
PL: Plumbagin
RCT: Reverse Cholesterol TransferRNA – Ribonucleic Acid
ROS: Reactive Oxygen Species
SD: Standard Deviation
siRNA: Small Interfering RNA
SR-B1: Scavenger receptor class B, Member 1
STR: Short Tandem Repeat
CETSA: Cellular Thermal Shift Assay
q.d.: *quaque die*
VLDL: Very Low Density Lipoprotein
