# Supplementary material for: Identification of CETP as a molecular target for estrogen positive breast cancer cell death by cholesterol depleting agents
Source: Genes Cancer. 2016 Sep;7(9-10):309–22. doi: 10.18632/genesandcancer.122 (PMC5115172; doi:10.18632/genesandcancer.122)
Supplement: Supplementary file 1 [file ganc-07-309-s001.pdf]

Identification of *CETP* as a molecular target for estrogen positive breast cancer cell death by cholesterol depleting agents – ESAU et al

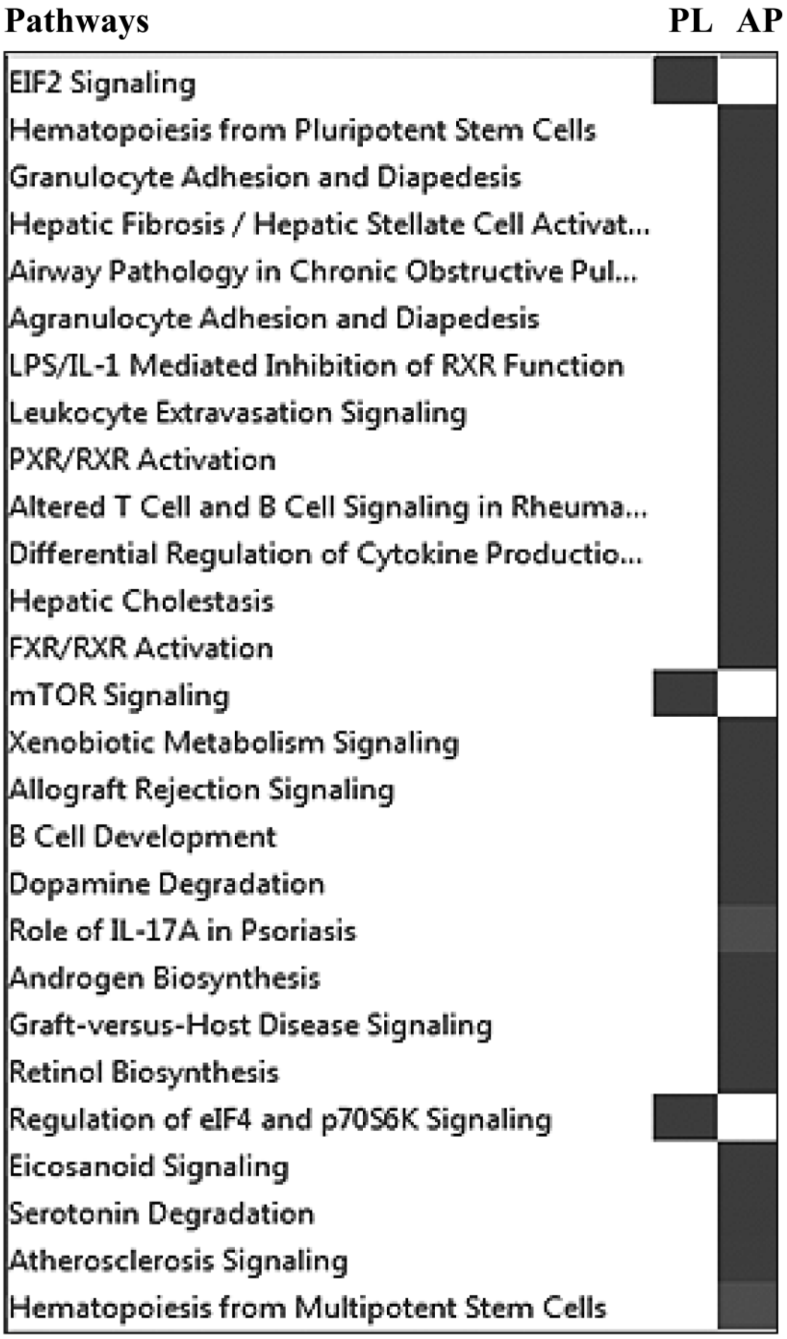

Supplementary Figure 1 (A) Heat map generated using IPA for downregulated pathways in 10 µM PL and AP treated MCF-7 cells.

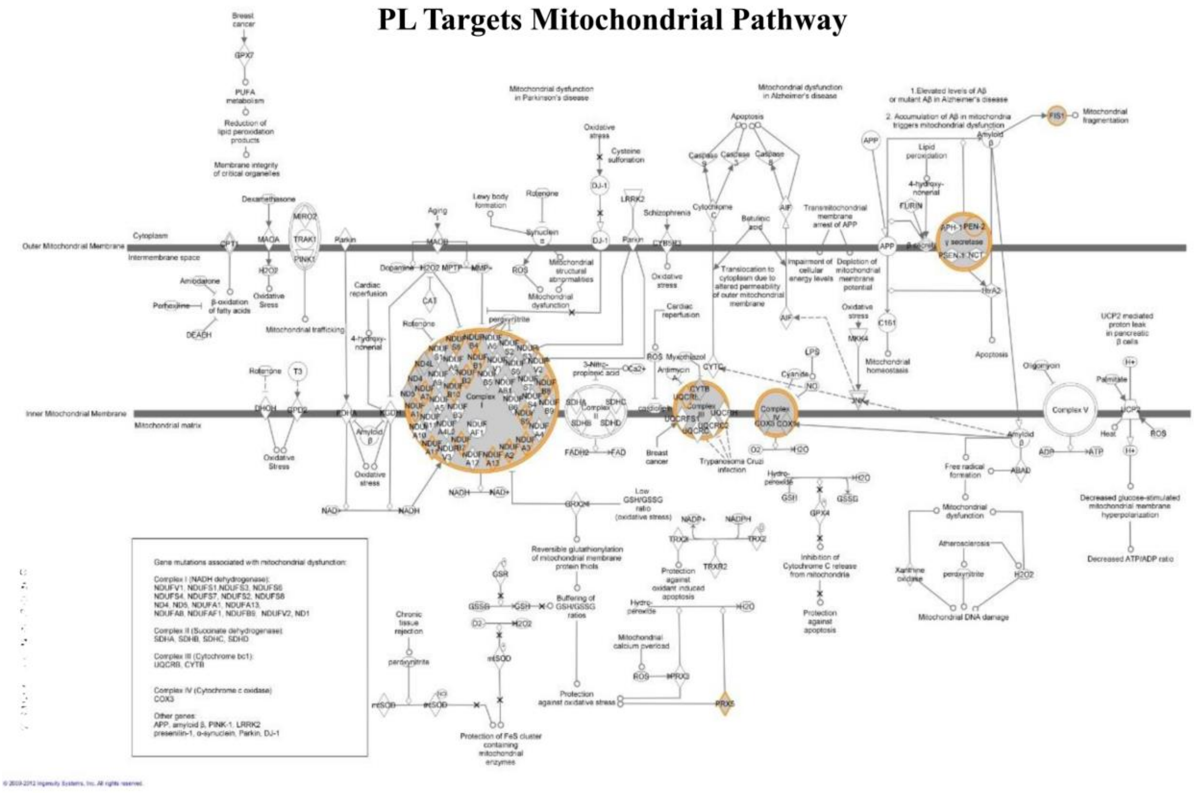

Supplementary Figure 2 PL responsive genes mapped to 'Mitochondrial Pathway'. MCF-7 cells treated with 10 µM PL for 6 h were subjected to microarray analysis. A cut off of 2 fold change in gene expression was applied and differentially expressed genes were mapped to pathways using IPA. Orange colour represents components of the mitochondrial pathway mapped by genes differentially expressed in response to PL treatment.

AP Targets Cholesterol Pathway

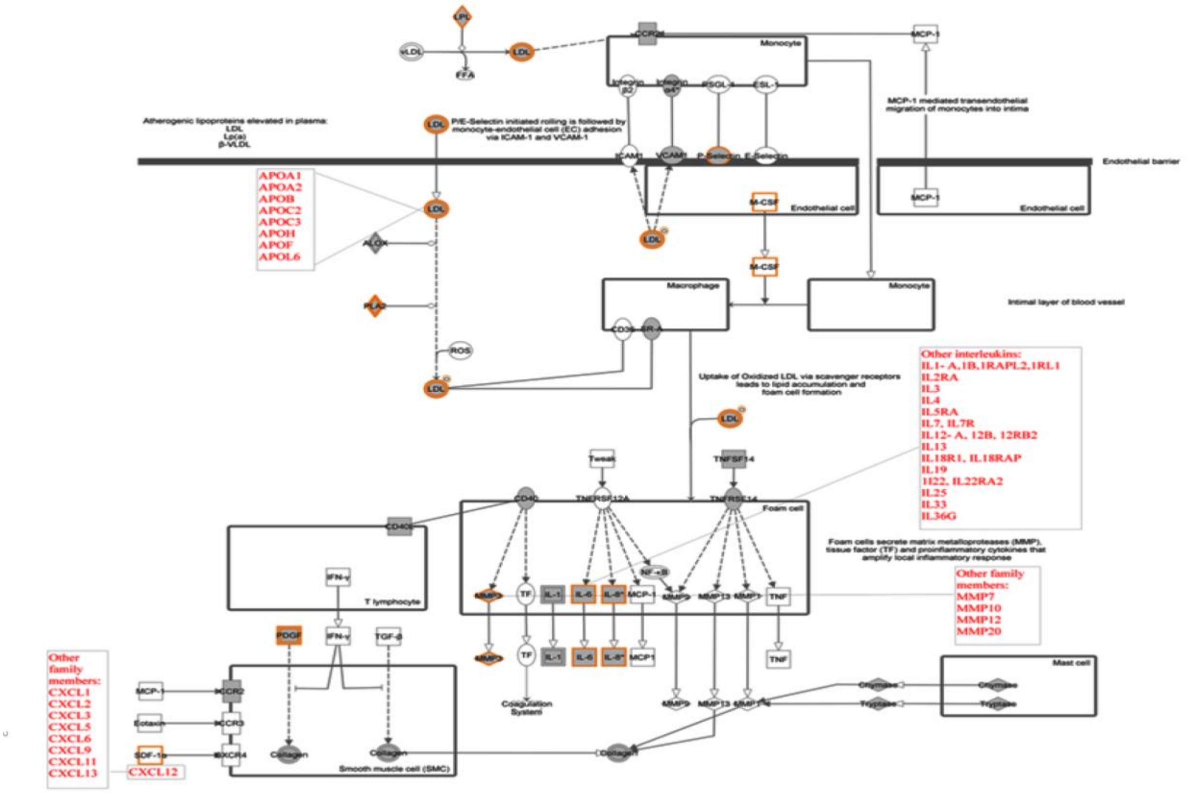

Supplementary Figure 3 AP responsive genes mapped to 'Atherosclerosis Signaling Pathway'. MCF-7 cells treated with 10 µM AP for 6 h were subjected to microarray analysis. Differentially expressed genes (2 fold change in expression) were selected and mapped to pathways in IPA. Orange colour boxes represent mapped genes. The lists of genes mentioned in 'red' are the symbols of genes present in the dataset in a gene family mapped to the pathway. Differentially expressed genes in AP treated cells mapped to the Cholesterol Pathway.

A

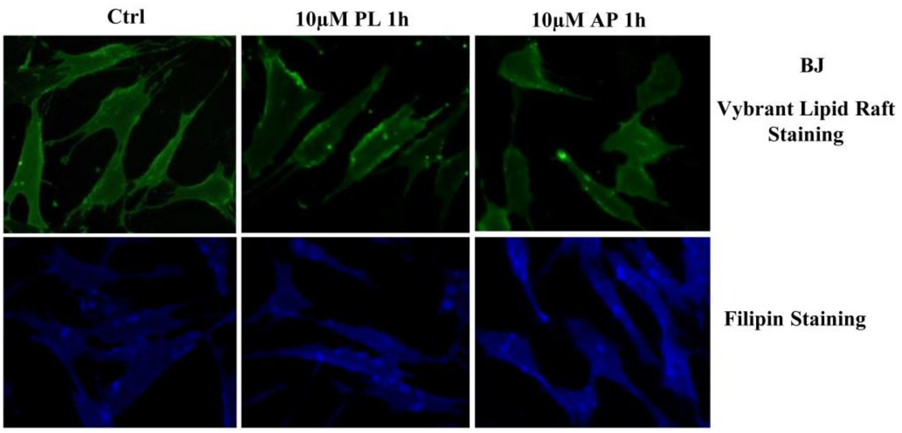

B

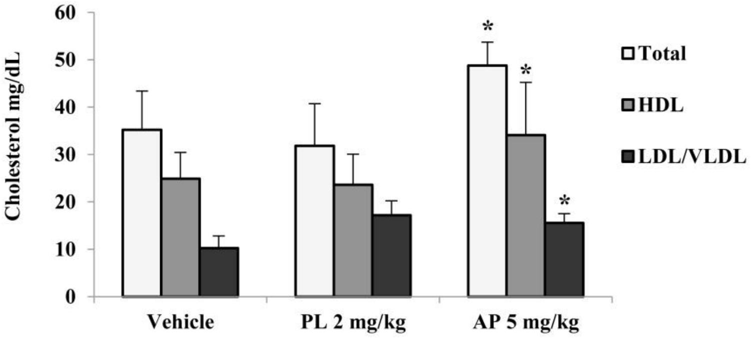

Supplementary Figure 4 (A) Lipid raft and filipin staining in BJ cells after treatment with 10 µM PL or AP for 1 h. Images are representative of three independent experiments (n = 3). **(B)** Levels of total cholesterol, HDL, and VLDL/LDL in serum of mice treated for 21 days with vehicle, 2 mg/kg PL or 5 mg/kg AP. Data shownas mean ± SD of vehicle group (n = 4), PL-treated (n = 4) and AP-treated (n = 5) groups ($P \leq 0.05$, *t*-test).

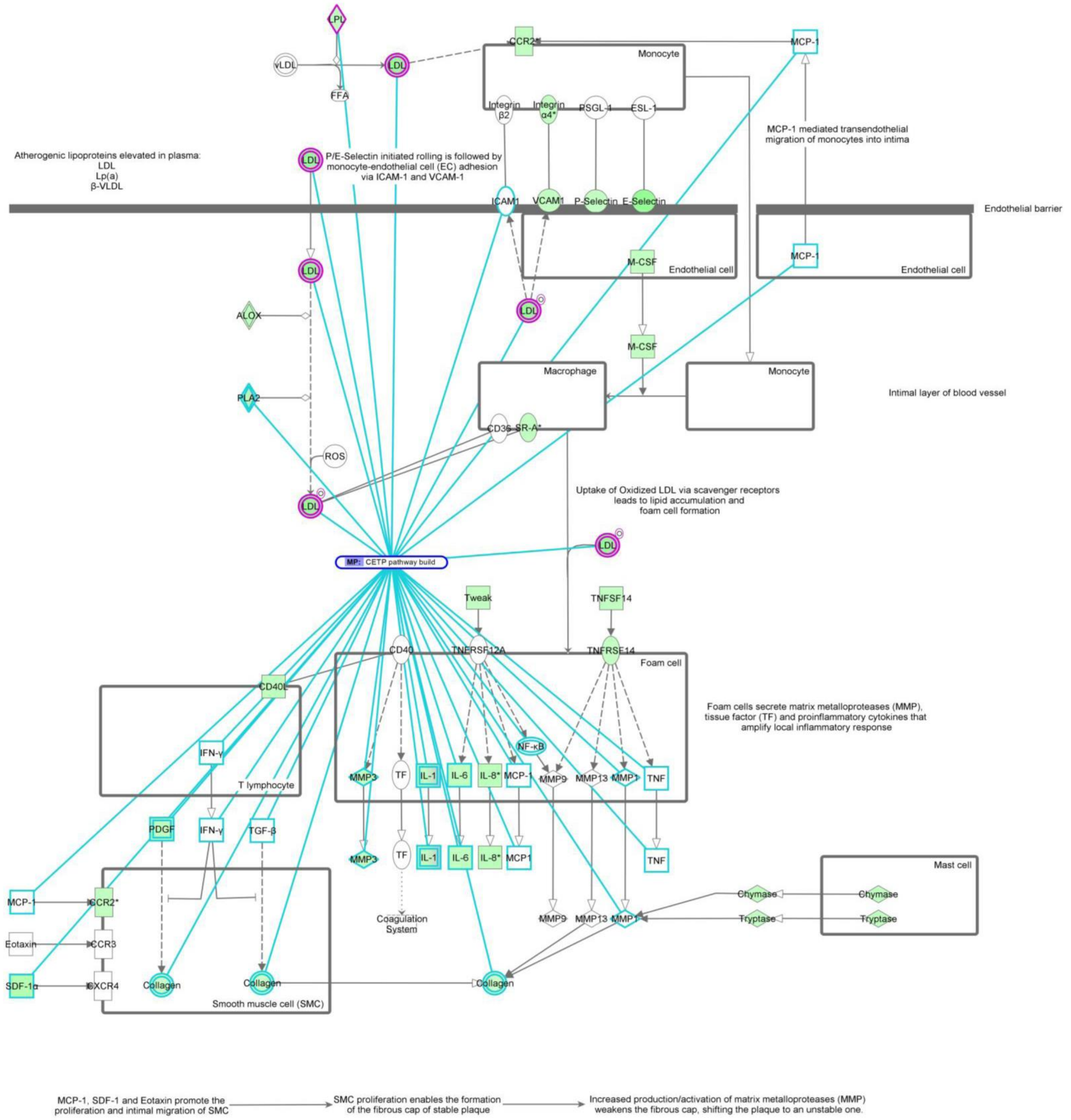

Supplementary Figure 5 CETP interacts with genes in the 'Atherosclerosis Signaling Pathway'. IPA was used to determine CETP interactions with genes involved in the atherosclerosis signalling pathway.

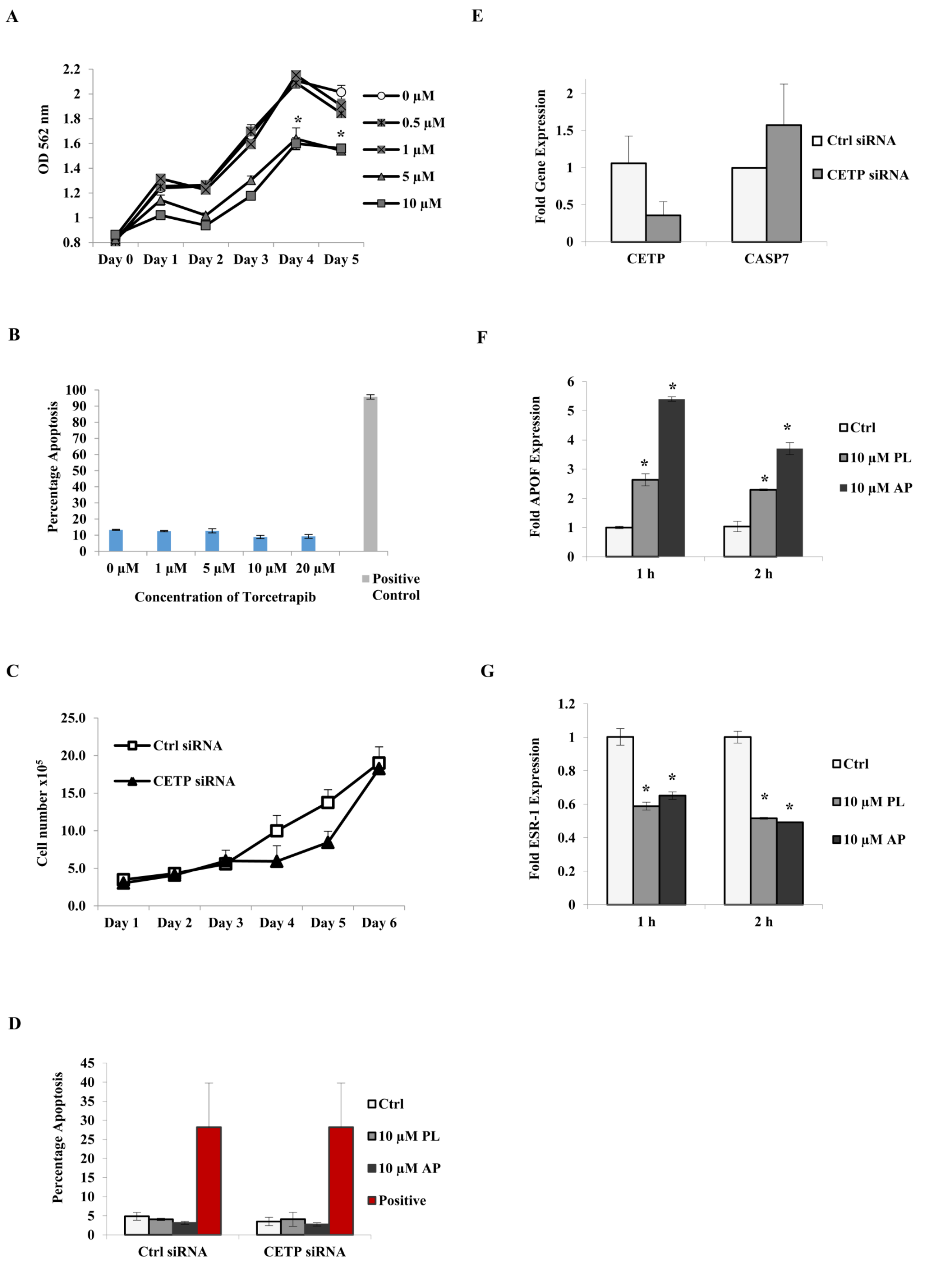

Supplementary Figure 6 (A and B) The effect of various concentrations of Torcetrapib on growth and apoptosis in MCF-7 ctrl and *CETP* siRNA knockout cells.**(C)** Cell number of BJ *CETP* siRNA and Ctrl siRNA cells over 6 days and (**D)** apoptosis in BJ ctrl siRNA and

CETP siRNA knockout cells treated with 10μM-PL or AP treatment. **(E)** *CETP* and Casp7 mRNA expression in MCF-7 ctrl and *CETP* siRNA cells 72 h after knockdown. **(F and G)** mRNA expression of APOF and ESR-1 in MCF-7 cells treated with 10μM-PL or AP for 1 and 2 h respectively. Data shown are representative of the mean ± SD of quadruplicate wells/condition in at least two independent experiments (n=2)(*P≤ 0.05, *t*-test).
